# The EMPCAN study: protocol of a population-based cohort study on the evolution of the socio-economic position of workers with cancer

**DOI:** 10.1186/s13690-019-0337-1

**Published:** 2019-03-20

**Authors:** Régine L. Kiasuwa-Mbengi, Victoria Nyaga, Renée Otter, Christophe de Brouwer, Catherine Bouland

**Affiliations:** 1 Belgian Cancer Centre, Department of Public Health and Epidemiology, Sciensano, Brussels, Belgium; 2 Research Centre for Environmental and Occupational Health, School of Public Health, Université Libre de Bruxelles, Brussels, Belgium

**Keywords:** Return-to-work, Cancer, Life course, Determining factors

## Abstract

**Background:**

The improvements in cancer control led to an increase in the number of cancer survivors, notably, in the working age population (16–64 years). There is a strong need to assess and understand their reintegration on the labour market, which underlines and ensures their social integration and quality of life. The objectives of the EMPCAN study is therefore to measure the scale of return-to-work after cancer and to identify the determining factors, allowing for the implementation of an adequate socio-professional support.

**Methods:**

We requested data from the Belgian Cancer Registry and the Crossroad Bank for Social Security. We included all socially insured Belgian workers diagnosed between 2004 and 2011 with colorectal, breast, head & neck, prostate, testis, lung and corpus uteri cancer. The end of (administrative) follow-up was 31st December 2012. We include demographic, health-related and work-related factors in the analysis and observed how these factors interplay to determine the working status. After having solved legal, ethical and technical issues for the coupling, we will perform survival analysis with competing risks using the Fine and Gray model; we will also perform a multistate model using transitions probabilities; and finally, a group-based modeling for longitudinal data using the ‘proc traj’ package in SAS.

**Discussion:**

The results of the EMPCAN study will allow the provision of an evidence-based support to professional reintegration policies. It will also bring some key features for the prediction of the cancer-related social security needs. Besides the raise of awareness among health professionals and policy makers, this study could lead to a better planning and organization of vocational rehabilitation programs.

**Electronic supplementary material:**

The online version of this article (10.1186/s13690-019-0337-1) contains supplementary material, which is available to authorized users.

## Background

Since two decades, improvements in cancer control led to an increased survival and subsequent prevalence of cancer survivors [1] with differences within and among European Members States [2]. In Belgium, 40% of the new cancer diagnoses are detected in the working age population (16–64) [3]. For this group, the ability to keep or resume work means maintaining the level of the household income, self-esteem, back to “normal”, to feel being cured, preservation of the quality of life, etc. [4–8].

However, the return-to-work (RTW) pathway is not obvious and encounters many challenges. It can imply temporary adjustments, different working capacities or new occupational aspirations [7–9].

The literature reports several obstacles to the RTW after cancer, directly related to the cancer and its treatments, but also depending on the work environment, personal beliefs and the reevaluation of the importance of working [10, 11]. In most European countries, authorities are reflecting on the legal and practical framework for the RTW after a long sickness absence [12].

At the moment in Belgium, no population-based study reports on estimates of the size of this phenomenon [13]. Worldwide, there have been some attempts to measure the rate of RTW after cancer [10, 14–16], mainly through cross-sectional studies, using samples of survivors from specific groups of cancer patients [7]. These studies report RTW rates varying between 40 to 80% [15].

The Cancer Centre of Sciensano, in collaboration with the School of Public Health of the Université Libre de Bruxelles designed and conducted the EMPCAN study. The objective of the current manuscript is to describe the design of the EMPCAN study which aims at quantifying the RTW after cancer. More specifically the objectives of EMPCAN are (1) to estimate the RTW rate after cancer diagnosis in Belgium; (2) to explore the differences in the rates and time to RTW according to demographic characteristics, cancer site, stage, treatment and type of employment and (3) to compare the rate of working cancer survivors with the general working population.

## Methods

### Description of the study

The EMPCAN study is a retrospective population-based cohort study, using data coming from three Belgian administrative registers. It includes workers aged 16–64 years, diagnosed in Belgium with breast, colorectal, lung, corpus uteri, prostate, head and neck or testis cancer between 1st January 2004 and 31st December 2011.

In the following, we describe (1) how we have decided on the variables to ask to the registers; (2) which procedures were followed to allow the linkage as well as the inclusion and exclusion criteria; (3) the list of variables included and (4) the type of analysis that we will perform.

### Study population and inclusion/exclusion criteria

The first inclusion criteria is the *age* (16–64 years old at the date of incidence) and the cancer-related information: the *date of diagnosis* (between 1st January 2004 and 31st December 2011) and the *type of cancer* (head and neck cancer C00-C14 and C30-C32; lung cancer C34; colorectal cancer C18-C20; breast cancer C50; corpus uteri C54; prostate C61 and testis cancer C62.

The second criteria concern the *working status* of the patient at the date of incidence and during the three preceding quarters. We excluded those patients who were long-term unemployed, disabled, handicapped or on sick leave at these dates.

### Study duration

The first cancer-related data available for the whole country started from 2004, the year which EMPCAN starts to include patients. As administrative health and social security registration in Belgium can have up to two years delay and that we started to design the EMPCAN study in 2015, we selected patients with a date of diagnosis between 1st January 2004 to 31st December 2011, so that the last who entered have at least 2 years of follow-up.

### Study flow

**Fig. 1:**
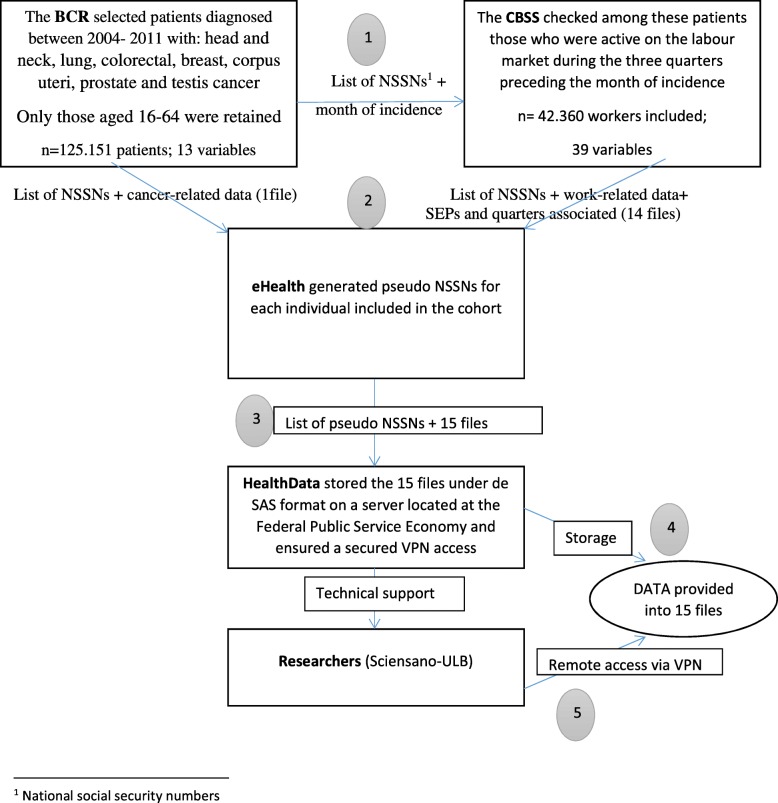
Dataflow for the coupling in the EMPCAN study, Belgium 2015–2020

The Fig. 1 presents the dataflow of the personal data from the providers to the researchers. The BCR started the process, with the selection of patients diagnosed from 1st January 2004 to 31st December 2011 included, with: head and neck cancer (C00-C14 and C30-C32); lung cancer (C34); colorectal cancer (C18-C20); breast cancer (C50); corpus uteri (C54); prostate (C61) and testis cancer (C62). Among them, the BCR only retained those aged 16–64 at the date of incidence. The list of national social security numbers (NSSN) of these patients was sent to the CBSS, which checked whether these patients were professionally active during the three quarters preceding^1^ the month of incidence. In line with privacy protection requirements and to ensure that the workers are unidentifiable, the BCR took a random sample of 75%.

The BCR and the CBSS sent, separately, the extracted data with the corresponding NSSNs to eHealth, via an electronic flow. The public institution eHealth is the only one authorized to proceed the linkage of data based on the NSSN. In our study, the main task of eHealth was to pseudo-anonymize the NSSNs, i.e. to generate new IDs for each NSSN and to keep the pseudo-anonymization key.

The pseudo-NSSNs and their associated data were transmitted by eHealth to HealthData.be which organized (1) the data storage on a secured server, (2) the transcription of the data into SAS data files and (3) a secured virtual private network (VPN) connection to the server for the researchers.

Finally, the researchers accessed the data that was provided through 12 SAS files with a total amount of 52 variables (Tables 1 and 2). Each file contains information related to a unique ID on individual level allowing retrieving data on the individuals in each file, along with the quarter corresponding to the socio-economic position observed (cross-sectional data). Each individual included can be observed for a maximum of 36 quarters (9 years).

**Table 1:** The list of demographic, cancer-related and work-related variables selected for the EMPCAN study, Belgium 2015–2020

Group of variables	Variables	Levels^a^	Primary source	Data provider
Demographic	Age (16–64)	11	NOSS^d^	CBSS^h^
	Gender	2	NR^e^	CBSS
	Region	5	NR	CBSS
	Civil status	12	NR	CBSS
Cancer-related	Cancer site	7	BCR^f^	BCR
	Stage	5	BCR	BCR
	Month of incidence^b^	96	BCR	BCR
	Treatment	4	IMA^g^	BCR
	Quarter of death	2	NR	CBSS
Work-related	Occupational class	5	NOSS	CBSS
	Code NACE2^c^ Code NACE3	84 352	NOSS	CBSS
	Salary class	8	NOSS	CBSS
	Work schedule	6	NOSS	CBSS
Personal-factors (not used)	Value of work			
	Self-assessed health status			
	Self-assessed work ability			
	Perceived quality of the relationship with colleagues			
	Perceived quality of the relationship with manager/head			

^a^the maximum number of values that the variable can take, as well as the primary source of information and the EMPCAN provider

^b^Date of the first microscopic (cytological or histological) confirmation of the malignancy

^c^Statistical Classification of Economic Activities in the EC. Accessible via: http://ec.europa.eu/eurostat/web/nace-rev2/overview (access on the 4th June 2018)

^d^National Office for Social Security

^e^National population registry

^f^Belgian Cancer Registry

^g^Inter-Mutuality Agency

^h^Crossroad Bank for Social Security

**Table 2:** The list of variables that relate to the socio-economic position within the Belgian social security system

Group of variables	Variables	Primary source	Data provider
Occupied	White collar	NOSS	CBSS
	Blue collar	NOSS	CBSS
	Self-employed	NOSS	CBSS
	Civil servant	NOSS	CBSS
Inactive	Occupational disease	FOD^a^	CBSS
	Occupational accident	FOI^b^	CBSS
	Sick leave	NIC^c^	CBSS
	Disability	NIC	CBSS
	Handicap	FPS SS^d^	BCR
Unemployment	Unemployment (job search)	NEO^e^	BCR
	Career break	NEO	BCR
	Social integration income	FPP SI^f^	BCR
	Pre-retirement	NEO	CBSS
	Entitled child	NOFA^g^	CBSS
	Exempt for employment search	NEO	CBSS
Other		CBSS	CBSS

^a^Fund for occupational diseases

^b^Fund for occupational injuries

^c^National InterMutualist College

^d^Federal Public Service for Social Security

^e^National Employment Office

^f^Federal Public Program for Social Inclusion

^g^National Office for family allowances

### Procedures for data collection

#### Selection of the determinants to investigate

In order to identify the determining factors of RTW after cancer, we conducted a literature review [11]. As the contextual factors (political and socio-economic) may be important in the RTW pathway [17–19], we presented the results to a group of field experts from different domains of expertise (oncology, occupational health, social security, academics, etc.) who confirmed and specified the determining role of each factor identified in the literature, into the Belgian context.

This exercise resulted in the identification of four groups of RTW determinants: socio-demographic, disease-related, work-related and person-related. Given the fact that the cohort that will be used will be selected within the cancer registry and that the study will use administrative data, the last domain, the person-related factors, cannot be investigated (Table 1).

**Fig. 2:**
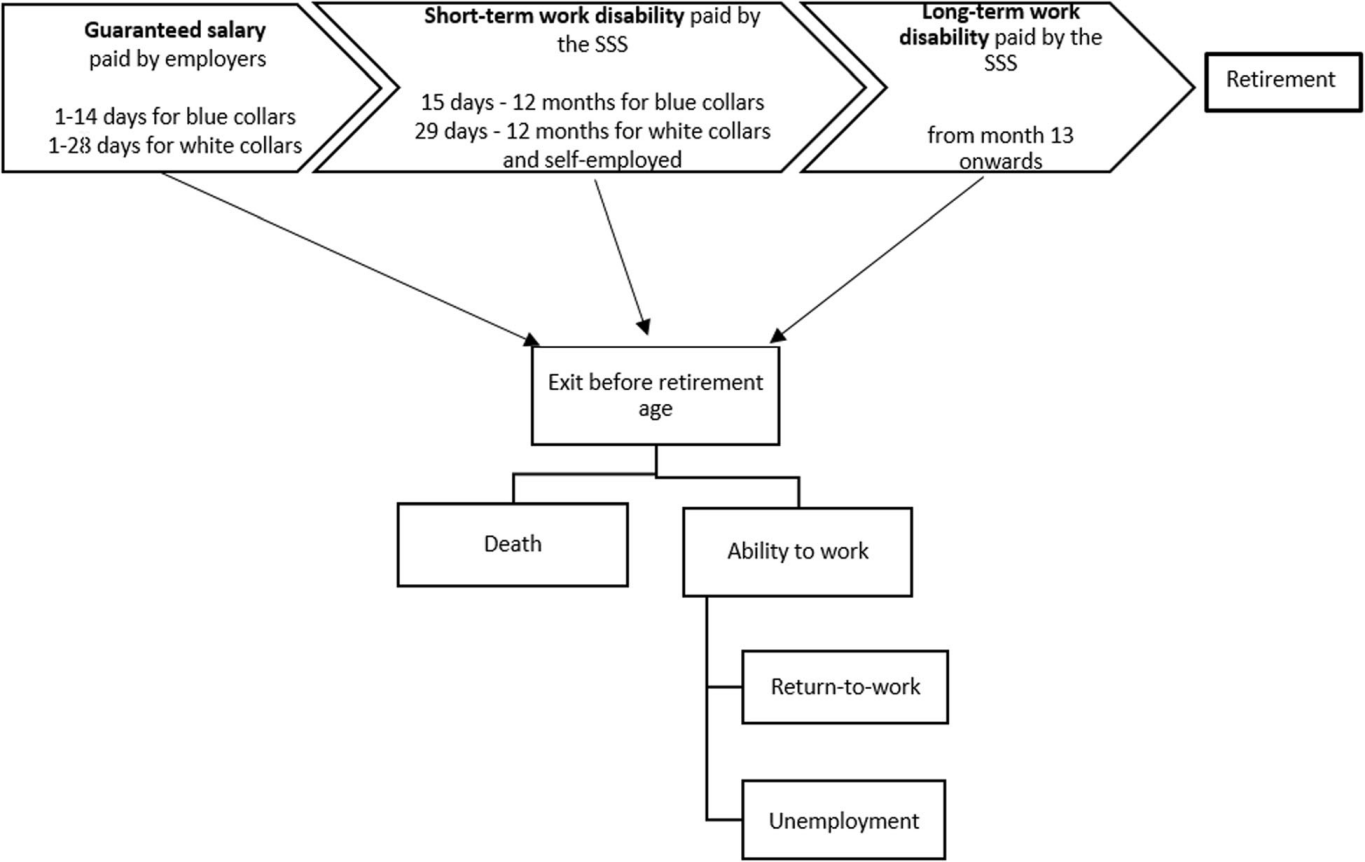
The Belgian social security scheme (SSS) related to sickness absence

After having identified and selected the determinants, we clarified the outcome (or main event of interest). According to the sickness absence literature [20], different outcomes are used to study the RTW after cancer [21, 22]: the ability to work, the ability to the return-to-work, the work performance, the job retention, the quality of life, etc. [7, 23]. In the EMPCAN study, the primary goal focuses on RTW after cancer. In the Belgian social security system, the working status refers to a socio-economic position, which is the position that a socially insured Belgian resident has on the labour market (Table 2). Hence, four main socio-economic positions are observed (Table 2): occupied (with four underlying classes), job seeker (with four underlying status), inactive (with five subgroups), and the others.^2^ The national social security scheme (SSS, Fig. 2) allows for the combination of up to three socio-economic positions at the same time, for one individual.

#### Identification of data sources and providers

Based on the list of variables that were identified, we looked into the Belgian healthcare registries to find from where the data could have been retrieved. Three main sources of information or registries were identified: (1) the Belgian Cancer Registry, (2) the Intermutality Agency and (3) Labour Market Datawarehouse.

**Fig. 3:**
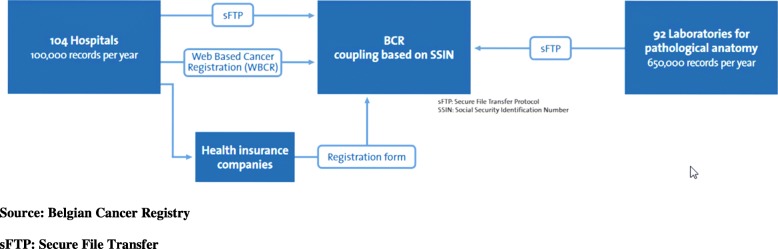
The cancer data collection of the BCR

For population-based cancer-related information, the Belgian Cancer Registry (BCR) was the most appropriate data source. The BCR collects information about all new diagnosed cancers in Belgium. The BCR receives cancer-related information from three sources: (1) the hospitals where (cancer) data managers are trained to retrieve the appropriate data; (2) the health insurance companies and (3) the laboratories performing the microscopic confirmation of malignancy (Fig. 3).

Based on these information, the BCR maps out the nature and extent of cancer in Belgium.

In addition, the cancer treatments were identified as important determining factors, having short, middle and long-term impact on the quality of life and health status of cancer survivors [24–28]. In Belgium, all treatments that are covered by the social security are registered by the health insurances, and transmitted to an overarching institution, the InterMutuality Agency (IMA). When it comes to cancer treatment, the BCR and the IMA have concluded an agreement to allow the BCR to couple all their cases with the treatment information of IMA.

Several social security institutions could provide us with the required data with regard to the socio-economic position and its underlying information. However, the Labour Market Datawarehouse (DWH MT&PS) was created in 1999, within the Crossroad Bank for Social Security (CBSS), with the aim to register and aggregate permanently administrative information of Belgian residents. Since 2003, 16 institutions send quarterly their information to the DWH MT&PS which has now information on 85% of the Belgian population.

As there was no precedent of such data coupling in Belgium, these different steps of coupling and controlling privacy, required more than two years to finally submit our demand to the national Privacy Commission, which provided its positive opinion in January 2016; this authorization started the data flow process among the registers and eHealth and the final files were made available for the researchers in October 2017 (Additional file 1).

### Data management and storage

According to the legal prescriptions and recommendations from the national Privacy Commission (based on the European Commission Communication [26]), the dataset resulting from the coupling of personal data coming from different registries has to be stored on a platform that is separated from those of the primary sources and a remote access has to be organized for the researchers to access the data.

For the EMCPAN study, this technical management (virtual private network connexion) is ensured by the HealthData.be service of Sciensano. In 2006, the national knowledge centre (KCE) [30] performed an inventory of the healthcare databases in the country, which revealed the existence of almost 150 different databases. In order to stimulate research, HealthData.be has been created, with the aim of standardization, homogenization and centralization of healthcare information, respecting the confidentiality principles for both patients and providers.

An additional requirement, in line with the ***proportionality principle,*** had to be foreseen: a small cell analysis will be conducted in order to balance the probability of person identification versus the level of detail of the data for the individuals included in the cohort.

### Data analysis

#### Exploratory data analysis

**Fig. 4:**
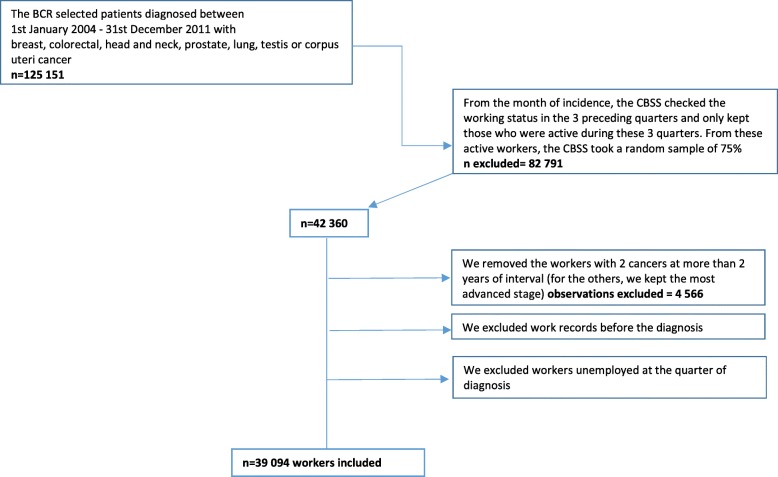
Data flowchart of inclusion and exclusion, EMPCAN study, Belgium 2015–2020

We will start with an exploratory analysis to understand the inclusion/exclusion flow, to clean the data (identify and exclude repetitions and inconsistencies, Fig. 4) and describe the main characteristics of the individuals included in the EMCPAN study.

The main objective is to explore the possible effect modification of variables (e.g. between cancer, stage and treatment) on the outcome.

#### Logistic regression

The logistic regression is often used for multivariate analysis purpose in epidemiology, to measure the association between event(s) of interest (dependent variables) and determining factors (independent variables). According to the main objectives of the EMPCAN study, we will use the logistic regression considering the (return to the) professional activity as the main event of interest, whether it is part-time or full-time. The determining factors are those presented in Table 1.

To ensure proper estimation of this event, we will have to take into account the probability of occurrence of other events, i.e. death and retirement. We will therefore use the Fine and Gray model [31] which allows for competing risks analysis and provision of cause-specific hazards.

Unlike in most Western countries, disability in Belgium is not an irreversible status and is therefore not granted until death or retirement. For that reason, disability is not considered as a competing event to work.

#### Multistate life tables (MSLT) and transitions among socio-economic positions

An important aspect of our data and event of interest is that workers can enter and exit the different socio-economic positions, over time. To capture this reality, the best method is to use a multistate model.

**Fig. 5:**
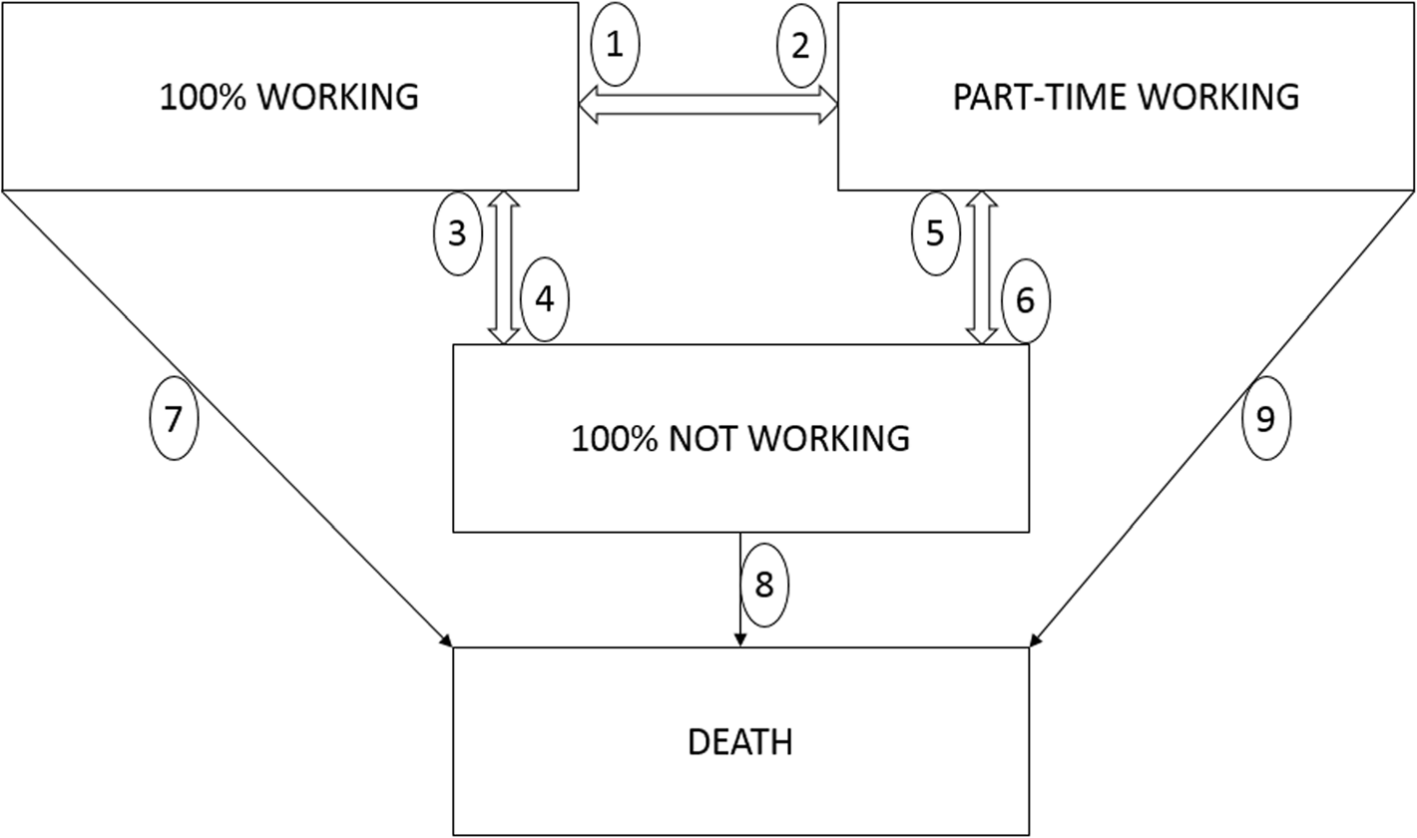
The four states and nine transitions of the Multistate model used in the EMPCAN study

If we consider the socio-economic positions as the states, we would have 14 different states in the model and 39 possible transitions. As our main interest is the working status, we reduced the model by focusing on four states: 100% working, 100% not working, part-time working and death (Fig. 5).

We will model these 9 possible transitions, among the four states and will incorporate covariates to explain (possible) differences among workers with cancer in the course of the follow-up.

#### Life course perspective

In addition of the transversal perspective and results that the logistic regression offers, we looked for a model allowing a longitudinal approach, emphasizing the employment trajectories and the transitions between the spectrum of the socio-economic positions (Table 2), among the workers with cancer.

For the trajectories, we will conduct a mixture modeling exercise that can be described as:

$$ T= quarter\ (q)12,q16,q20 $$

$$ {y}_{it}=\left\{\begin{array}{c}1\  if\ employed\ in\ quarter\ t\\ {}0\  if\ unemployed\ in\ quarter\ t\end{array}\right. $$

$$ {\boldsymbol{y}}_i=\left\{{y}_{i1},{y}_{i2},\dots, {y}_{iT}\right\} $$

$$ f\left({\boldsymbol{y}}_i|{\boldsymbol{z}}_i,{w}_i\right)=\sum \limits_j^JP\left({C}_i=j|{\boldsymbol{Z}}_i={\boldsymbol{z}}_i\right)P\left({\boldsymbol{Y}}_i={\boldsymbol{y}}_i|{C}_i=j,{\boldsymbol{W}}_i={\boldsymbol{w}}_{\boldsymbol{i}}\right) $$

$$ P\left({C}_i=j|{\boldsymbol{Z}}_i={\boldsymbol{z}}_i\right)=\frac{e^{\theta_j+{\boldsymbol{\lambda}}_j{z}_i}}{\sum_1^J{e}^{\theta_j+{\boldsymbol{\lambda}}_j{z}_i}} $$

$$ P\left({\boldsymbol{Y}}_i={\boldsymbol{y}}_i|{C}_i=j,{\boldsymbol{W}}_i={\boldsymbol{w}}_{\boldsymbol{i}}\right)=\frac{e^{\sum_{o=0}^O{\beta}_o^j{Q}_{it}^o+{w}_{it}{\delta}_j}}{1+{e}^{\sum_{o=0}^O{\beta}_o^j{Q}_{it}^o+{w}_{it}{\delta}_j}} $$

$$ O=0,1,2,3,4 $$

where:

*x*_*i*_ time-stable predictor variables.

*R*_*it*_ time-dependent variables.

Up to the polynomial of order 4 due to software limitations.

An important step of the modeling is the model selection for which two aspects are assessed: the optimal number of components and the appropriate degree of the polynomial modeling each group trajectory. The Bayesian information criterion (BIC) will be used to select the optimal model. It can be used to assess the fit of both nested and unnested models under fairly general circumstances.

The best model will have the maximum BIC i.e. the least negative value.

The SAS procedure Proc Traj command computes the BIC as follows:

$$ BIC=\log (L)-0.5\ast \log (n)\ast k, $$

where, L is the maximized value of the log likelihood, n is the sample size and k is the number of parameters in the model.

### Legal and ethical approval

#### Legal and technical requirements

The EMPCAN study has been designed by the Cancer Centre of Sciensano in collaboration with the Research Centre for Environmental and Occupational Health of the School of Public Health of the Université Libre de Bruxelles. Both institutions are owners of the results.

As prescribed by the European Commission Communication of 2012 [29], the exchange of health-related personal data has to be supported and organized by eHealth services. These governmental institutions play the important role of the third trusted party in charge of bringing personal data from different sources together.

#### Legal and ethical issues for coupling personal data

The coupling of personal data performed in the EMCPAN study has been realized before that the 2018 EU *General Data Protection Regulation (GDPR) came into force. The legal framework in which this linkage has been done, relates on four main legal bases presented in the* Table 3*.*

**Table 3:** The legal bases used and followed for the linkage of personal data within the EMPCAN study, Belgium 2015–2020

Legal text	What?	Parties concerned
Art. 39 of Chapter VI of the Law of 13th December 2006	Use of the national social security number to identify cancer patients	BCR
Art. 39 of Chapter VI of the Law of 13th December 2006	The right to register cancer and cancer-related information	BCR
Art. 39 of Chapter VI of the Law of 13th December 2006	To transfer cancer-related coded data for research purpose	BCR
Art. 4 – Chapter II of the Law of 8th December 1992	Definition of the conditions to allow the treatment of personal data	Researchers
Art. 7 – Chapter II of the Law of 8th December 1992	Definition of the conditions to allow the treatment of health-related personal data	Researchers
Art 5 – Chapter III of the Law 21st August 2008	Mission of the eHealth platform	eHealth (governmental third trusted party)
Art. 16 – Chapter I of the Law 10th April 2010	The right to build and use databases for scientific purpose	Sciensano (HealthData.be and Cancer Centre)

The execution of this linkage has been discussed with the two main data providers (BCR and CBSS) and HealthData.be, first separately. We presented the main objectives of the study, discussed the data required and we asked for the internal legal, ethical and technical requirements that we had to meet (e.g. the presentation of the project to internal boards).

Second, several preparatory meetings took place with all the partners in order to discuss and ensure the technical and ethical feasibility of the study.

The main points under discussion concerned the definition of the cohort (inclusion and exclusion criteria) and the choice of the variables which were the most appropriate regarding the study objectives, i.e. the discussion of the proportionality principle.

For the technical and ethical aspects, we received advices and recommendations of lawyers from the governmental platform eHealth. Their two main concerns were: (1) the fact that the final coupled dataset has to be stored on a completely separated server and that (2) the data holder has to foresee access for the researchers via a virtual private network (VPN) connexion.

## Publication plan

Besides this protocol, at least three publication in peer reviewed journals are foreseen. A first paper with the description of the cohort and the results of the logistic regression. A second paper will focus on the lifecourse perspective and the trajectories observed among the workers included in the study. A third paper will concern the multistate model and the transition probabilities among the different socio-economic positions.

### Strengths and limits of the EMPCAN study

The main strength of the EMPCAN study is its representativeness. A recent review on quantitative studies on RTW after cancer [15] reports that only one on the twelve included studies was population-based. The main reason is probably that the coupling of health, socio-economic and administrative data requires many legal and ethical steps. Another one is that most of these data are not available neither accessible at national level.

The main limit of EMPCAN is that it doesn’t include three important aspects to completely (comprehensively) understand and explain the RTW after cancer. First, the patient’s experience and self-assessment of their health status and (new) occupational aspirations are not captured in administrative data. Second, we do not include the employer’s perspective with their ability and willingness to reintegrate workers with cancer. Thirdly, cancer-related information is provided at baseline as the treatments are those provided during the first twelve months following the month of incidence, which limits the possibility to appreciate the health status of the workers on the following years on which he is observed. Similarly, cancer treatment related or preexisting comorbidities could be of high importance in the ability to work but are currently missing in our data.

However, in a previous study, we build a proxy for the health status, using the relative survival corresponding to the cancer site [32]. This exercise could also be performed in EMPCAN, using in addition of the relative survival, the stage at diagnosis and the treatments received.

## Discussion

The EMPCAN will bring important insights on the RTW after cancer and will allow the identification of the determining factors to be considered for the development of occupational rehabilitation interventions. The combination of the results from both cross-sectional and longitudinal approaches will allow a better understanding and explanation of the changes and stability of the socio-economic positions in the years after the cancer diagnosis. This should provide elements for the identification of impediments or facilitators that relate to the social security scheme.

Eventually, the more specific needs and remaining gaps in the knowledge regarding the RTW after a long sickness absence will be identified and suggested as future research perspectives.

## Additional file

Additional file 1: List of files and variables available in the EMPCAN database. (DOCX 18 kb)

## Abbreviations

BCR: Belgian Cancer Registry
BIC: Baysian Information Criterion
CBSS: Crossroad Bank for Social Security
DWH MT&PS: Datawarehouse Marché du travail & Protection sociale (Datawarehouse Labour Market & Social Protection)
FOD: Funds for Occupational Diseases
FPP SI: Federal Public Program Social Inclusion
FPS SS: Federal Public Service Social Security
GDPR: General Data Protection Regulation
IMA: InterMutuality Agency
KCE: Knowledge center of expertise
NEO: National Employment Office
NIC: National InterMutualist College
NOFA: National Office for Family Allowances
NOSS: National office for Social Security
NR: National population Registry
NSSN: National social security number
RTW: Return-to-work
SSS: Social Security Scheme
VPN: Virtual Portal Network

## Footnotes

1 The available information at the CBSS corresponds to the socio-economic position(s) of the socially insured individual at the last day of the quarter.

2 This group mainly include cross-border and international workers, seamen and housewives/men.
