# Supplementary material for: The EMPCAN study: protocol of a population-based cohort study on the evolution of the socio-economic position of workers with cancer
Source: Arch Public Health. 2019 Mar 20;77:15. doi: 10.1186/s13690-019-0337-1 (PMC6427850; doi:10.1186/s13690-019-0337-1)
Supplement: Supplementary file 1 — List of files and variables available in the EMPCAN database. (DOCX 18 kb) [file 13690_2019_337_MOESM1_ESM.docx]

## List of files and variables available in the EMPCAN database

| ***name variable*** | ***explanation*** | ***time-related  (varying)*** |
| --- | --- | --- |
| **file ACTIV** | *provide information on each quarter during which the individuals are working* |  |
| **IDC_PAT** | ID of the patient, common to each file | no |
| **CD_JOB_TPE** | occupational class of the worker; it has 4 levels | no |
| **CD_WORK_SCHED** | type of working time/schedule; it has 6 levels | yes |
| **TX_PT_CLASS** | percentage of part-time; it has 13 levels | yes |
| **TX_SLRY_CLASS** | class of salary ; it has 8 levels | yes |
| **CD_NACE_3** | type (sector of the) company in which the worker works; it has 352 levels | yes |
| **CD_NACE_2** | type (sector of the) company in which the worker works; it has 84 levels | yes |
| **CD_JOB** | the code of the occupation that is related to the disease (only available  for those cancer which are occupation-related) | no |
| **TX_PERIOD** | the quarter to which the information contained in the line correspond;  it has 35 levels |  |
| **file CANCER** | *provide cancer and treatment-related information* |  |
| **IDC_PAT** | ID of the patient, common to each file | no |
| **TX_CAN_TPE** | cancer site; it has 7 levels | no (but relapses) |
| **TX_CAN_STG** | stage of the disease at the date of incidence | no |
| **TX_YR_MTH** | month of incidence (when the cancer is discovered) | no |
| **FL_CHEMO_12M** | chemotherapy received on the 12months following the date of incidence | no |
| **FL_HORMO_12M** | hormonotherapy received on the 12months following the date of incidence | no |
| **FL_IMUNO_12M** | imunotherapy received on the 12months following the date of incidence | no |
| **FL_RADIO_12M** | radiotherapy received on the 12months following the date of incidence | no |
| **file DCD** | *provide the quarter of death for those who died…* |  |
| **IDC_PAT** | ID of the patient, common to each file |  |
| **TX_YR_QTR** | the quarter when the patient died | no |
| **file FBZ** | *provide information on those with an occupational disease* |  |
| **IDC_PAT** | ID of the patient, common to each file |  |
| **CD_JOB** | The code of the occupation that caused the disease |  |
| **CD_PATHO** | The code of the disease (see sheet values) |  |
| **NR_YEAR_DCSN** | the year of decision |  |
| **NR_YEAR_INCAP_STRT** | the year of start incapacity |  |
| **TX_CAT_PRCNT** | the percentage of work incapacity related to the occupational disease |  |
| **TX_PERIOD** | quarter associated with the status "affected by an occupational disease" | yes |
| **file FODSZ** | *provide information on those who are handicapped* |  |
| **IDC_PAT** | ID of the patient, common to each file |  |
| **TX_HANDICAP_PRCNT** | percentage of the handicap |  |
| **TX_PERIOD** | the quarter associated with the status "handicapped" |  |
| **file LFL** | *provide information on those who receive social integration income (OCMW-CPAS)* |  |
| **IDC_PAT** | ID of the patient, common to each file | no |
| **TX_PERIOD** | the quarter associated with receiving the social integration income | yes |
| **file LPB** | *provide information on those who receive unemployment benefits* |  |
| **IDC_PAT** | ID of the patient, common to each file | no |
| **CD_REAS_BREAK** | why they take a career break: never provided |  |
| **TX_PERIOD** | the quarter associated with receiving the social integration income |  |
| **file NIC** | *information on those who receive social benefits from the sickness insurance* |  |
| **IDC_PAT** | ID of the patient, common to each file | no |
| **MS_DAYS** | number of days that they receive(d) the social benefits | yes |
| **CD_NAT_BENEF** | type of benefits | yes |
| **TX_PERIOD** | the quarter corresponding at the reception of social benefits from the SI |  |
| **file PERS** | *provide demographic information* |  |
| **IDC_PAT** | ID of the patient, common to each file | no |
| **CD_SEX** | gender | no (normally not) |
| **TX_LIPRO** | marital status and presence of children (see sheet values) | yes |
| **TX_AGE_CLASS** | 5 year class of age; it has 11 levels | yes |
| **TX_REGION** | region where they live; it has 5 levels | yes |
| **TX_PERIOD** | quarter at which the information is observed |  |
| **file RIZIV 1** | *provide information on those who are disabled* |  |
| **IDC_PAT** | ID of the patient, common to each file | no |
| **CD_ILLNESS** | code associated with the illness having caused the disability | yes |
| **TX_PERIOD** | quarters during which the patient has been recognized as disabled |  |
| **file RIZIV 2** | *provide information on those who are disabled* |  |
| **IDC_PAT** | ID of the patient, common to each file |  |
| **CD_RETIRMENT** |  |  |
| **NR_YR_MNTH_ILLNESS** | month and year of… (start of the disease) |  |
| **TX_PERIOD** | quarters during which the patient has been recognized as disabled |  |
| **file SOCECO** | *provide information the reasons for not being working* |  |
| **IDC_PAT** | ID of the patient, common to each file |  |
| **FL_ACTIV** | is the individual actively working | yes |
| **FL_JOB_SRCH** | is the individual looking for a job (unemployed) | yes |
| **FL_OTHER** |  | yes |
| **FL_CARR_PAUSE** | is the individual having a career break | yes |
| **FL_EXEMPT_UNEMP** | is the individual exempted of looking for a job | yes |
| **FL_SOC_SUPT** | is the individual receiving social integration income | yes |
| **FL_BRIDGE_PENSION** | is the individual pre-retired | yes |
| **FL_ENTITL_CHLD** | is the individual a depending child | yes |
| **FL_DISAB_NSUR** | is the individual receiving social benefits from the sickness insurance | yes |
| **FL_INVALID** | is the individual disabled | yes |
| **FL_WORK_ILLN** | does the individual have an occupational disease | yes |
| **TX_PERIOD** | quarter at which the information is observed |  |
| **file WERKL** | *provide information the unemployment* |  |
| **IDC_PAT** | ID of the patient, common to each file |  |
| **NR_DURATION** |  |  |
| **NR_PWA_HRS** | number of hours worked | yes |
| **NR_DAYS** | number of days for which they received social benefits | yes |
| **TX_AMT_CAT** | class-level of the social benefits received | yes |
| **TX_PERIOD** | Quarter at which the information is observed |  |
